# Attitudes toward clinical trials across the Alzheimer’s disease spectrum

**DOI:** 10.1186/s13195-017-0311-5

**Published:** 2017-10-04

**Authors:** Michelle M. Nuño, Daniel L. Gillen, Kulwant K. Dosanjh, Jenny Brook, David Elashoff, John M. Ringman, Joshua D. Grill

**Affiliations:** 1 Institute for Memory Impairments and Neurological Disorders, University of California, Irvine, CA USA; 2 Department of Statistics, University of California, Irvine, CA USA; 3 Division of General Internal Medicine/Health Services Research, David Geffen School of Medicine at the University of California, Los Angeles, CA USA; 4 Department of Medicine, University of California, Los Angeles, CA USA; 5 Department of Neurology, University of Southern California, Los Angeles, CA USA; 6 Department of Psychiatry and Human Behavior, University of California, Irvine, CA USA

**Keywords:** Alzheimer’s disease, Clinical trials, Recruitment, Mild cognitive impairment, Preclinical

## Abstract

**Background:**

Research has revealed that manifest Alzheimer’s disease (AD) dementia is preceded by preclinical and prodromal phases during which pathology is accumulating but function remains intact. This understanding and concern that disease-modifying interventions initiated at the dementia stage may come too late in the neurodegenerative process to be successful has led to a paradigm shift in AD clinical trials. AD trials now enroll patients with mild cognitive impairment (MCI) and persons with no cognitive symptoms. Trial designs are similar to those enrolling dementia participants. We set out to test the hypothesis that attitudes towards trial design features differ among different potential AD trial populations.

**Methods:**

We sent a survey composed of 37 items assessing specific trial elements to 246 cognitively normal, MCI, and AD dementia participants at the University of California Los Angeles (UCLA) Alzheimer’s Disease Research Center (ADRC), from whom we received 91 responses (37 cognitively normal, 32 MCI, and 22 dementia). To quantify willingness to enroll, we created three composite scenarios by summing responses and fitting proportional odds models with a binary outcome variable for whether patients were highly willing to participate in low-, moderate-, or high-risk and burden trials.

**Results:**

MCI participants less frequently correctly self-identified their diagnoses than those with dementia or normal cognition. Compared to dementia patients, the odds of participating in a low-risk, low-burden trial were 12% lower for MCI patients (odds ratio (OR) = 0.88, 95% confidence interval (CI) 0.23–3.29) and 70% lower (OR = 0.30, 95% CI 0.08–1.09) for cognitively normal participants. With increasing risk and burden, willingness to enroll decreased and the gap in relative willingness between diagnostic groups increased. In the medium-risk, medium-burden scenario, the estimated OR was 0.64 (95% CI 0.17–2.40) for MCI and 0.21 for the cognitively normal (95% CI 0.06–0.77). In the high-risk, high-burden scenario, the estimated OR indicated reduced willingness for MCI (OR = 0.27, 95% CI 0.06–1.15) and cognitively normal respondents (OR = 0.12, 95% CI 0.03–0.54).

**Conclusions:**

These results suggest that AD trials enrolling predementia populations, especially those requiring frequent visits and implementing biomarker testing procedures, may encounter challenges to enrollment.

**Electronic supplementary material:**

The online version of this article (doi:10.1186/s13195-017-0311-5) contains supplementary material, which is available to authorized users.

## Background

Alzheimer’s disease (AD) is a progressive neurodegenerative disorder and the most common cause of dementia. A few symptomatic therapies are approved for AD but, as yet, no therapy has been successfully demonstrated to slow the cognitive and functional declines that characterize the disease [1]. Myriad challenges to developing disease-modifying therapies for AD exist. Among these, numerous studies indicate that by the time dementia is diagnosed neurobiological changes have been occurring for a decade or longer [2]. Based on this premise, research diagnostic criteria for mild cognitive impairment (MCI) due to AD [3] and prodromal AD [4, 5], as well as preclinical [6] or asymptomatic AD [4, 5], have been proposed for use in earlier disease clinical trials of potential disease-modifying therapies.

A key determinant of clinical trial success is the timely recruitment of eligible participants [7]. AD dementia clinical trials face a multitude of barriers to successful recruitment and often encounter slow or inadequate accrual [8]. While the barriers to AD dementia trial recruitment have been moderately well characterized [9–16], fewer studies explore trial decision-making in MCI [17–19] or preclinical AD [20, 21]. It will be important to understand how these populations differ in their approach to trial decisions, given that many aspects of AD trial designs, such as the outcomes used to assess efficacy, the incorporated biomarkers, and the requirement of a study partner, have been held constant as the field has evolved to include earlier and earlier disease participants. In fact, a number of candidate therapies, some with associated risks such as vasogenic edema or cerebral hemorrhage, are under simultaneous investigation in two or three AD diagnostic categories.

To begin to elucidate whether and how barriers to trial recruitment differ with disease stage, we sent a single survey instrument to cognitively normal, MCI, and AD dementia longitudinal observational research participants. We hypothesized that attitudes toward trial designs would differ and willingness to participate would decrease in less clinically affected persons.

## Methods

### Participants

Participants for this study were recruited from the longitudinal cohort study within the University of California Los Angeles (UCLA) Alzheimer’s Disease Research Center (ADRC). We included participants from three diagnostic groups who had consented to be contacted about additional studies: cognitively normal, MCI, and AD dementia. Patients were excluded from the study if they were determined to have dementia stemming from a non-AD etiology, did not speak English fluently, did not attend a follow-up visit at the UCLA ADRC in the 12 months preceding the survey, or had previously enrolled in a clinical trial. After applying these exclusion criteria, the survey was sent to 246 eligible participants.

### Data collection

A packet was mailed to eligible participants that included the survey, a brief introduction to the goals of the study, an addressed stamped return envelope, and a consent form. To increase the response rate, we telephoned participants who had not returned completed surveys 1 month after dissemination.

The data collection instrument consisted of 37 forced choice questions. The full survey is included in Additional file 1: Appendix. The survey was created based on previous interview studies focusing on understanding barriers to participation in AD prevention clinical trials [20]. Initial survey items asked participants to self-identify as someone with AD dementia, MCI, or normal cognition and relate these diagnoses to AD trial constructs (e.g., normal participants should approach the survey by considering enrolling in an AD prevention trial, MCI participants should consider enrolling in a trial to lower the risk of dementia, and AD dementia patients should consider a clinical trial of a medication to slow the progression of the disease). To better contextualize responses, the initial survey also asked the extent to which a participant’s study partner assisted them in completing the survey (no help, help with less than half the questions, help with more than half the questions, completed in partnership, or completed on behalf of the study participant by the study partner). Remaining survey items investigated participants’ willingness to enroll in clinical trials with different attributes as well as their reasons for enrolling in such studies. Survey items examined specific trial lengths and visit frequencies, varying study procedures (e.g., magnetic resonance imaging (MRI), positron emission tomography (PET) scans), candidate therapies of different modes of administration (e.g., diet, pill, vaccines), and possible side effects (e.g., headache, bleeding in the brain or gut). Most questions elicited responses on a seven-point Likert scale in which “1” represented “Disagree very much” or “Extremely unlikely”, “4” was “Neutral”, and “7” was “Agree very much” or “Extremely Likely.”

### Data analysis/statistical methods

The responses from each participant were linked to their demographic and clinical data from the UCLA ADRC database, including their research consensus diagnosis. Patient demographic data included race, gender, marital status, and living situation. The living situation of a respondent was categorized as living alone, with a spouse, with a relative or friend, or with a group. The patient sample was characterized using summary statistics, including the mean and standard deviation for quantitative variables and the count and frequency for categorical variables, stratified by clinical diagnosis.

To investigate agreement between ADRC consensus and self-reported diagnoses among those with both data points, we used unweighted and weighted kappa statistics [22, 23].

To approximate willingness to participate in trial scenarios with multiple attributes, we a priori created three composite scores by summing selected responses. The questions selected for the composite scores are based on the literature [8, 16] as well as practical experience. In the low-risk, low-burden scenario, we summed responses for 2-year clinical trials, oral experimental medications, annual visits, and trials that included MRI. For the medium-risk, medium-burden scenario, we considered responses for 5-year clinical trials, oral experimental medications, monthly follow-up visits, and trials that involved PET. Finally, the high-risk, high-burden scenario composite consisted of responses for 5-year clinical trials, infused experimental medications, weekly visits, and the use of lumbar puncture. Because the scores for each question ranged from 1 to 7, composite scores ranged from 5 to 35 for each scenario. We calculated Cronbach’s alpha for the risk and burden constructs in each scenario to investigate internal consistency. All statistics ranged from 0.73 to 0.91, indicating acceptable to excellent consistency.

To investigate differences in willingness to enroll by clinical diagnosis, we fitted a proportional odds model (ordinal logistic regression) for each scenario where the response was a discretization of the composite willingness score in each case (low risk and burden, medium risk and burden, and high risk and burden). The discretization of willingness for each of the three scenarios was derived using breakpoints at 21 and 28, which were selected a priori; > 21 represents above neutral willingness and > 28 represents high willingness. We chose to not use data-driven cutpoints in an effort to minimize potential inflation of type I errors. We a priori chose adjustment variables (gender, education, age, partner involvement in completing the survey, and marital status) in the model based upon their potential to confound the association between dementia status and trial willingness [8]. The proportional odds model assumes that the odds ratio (OR) for a “high” response that is associated with a given covariate is constant regardless of where the breakpoint that defines a “high” response lies (either above 21 or above 28 in this case). Thus, this approach avoids assumptions regarding the relative spacings of the cutpoints. The proportional odds assumption was assessed by fitting two separate logistic regression models (dichotomizing at greater than 21 and at greater than 28, respectively) and comparing the coefficients associated with each covariate across the two models. This investigation did not yield evidence that the proportional odds assumption was violated.

Missing responses were present in some completed surveys and ranged from 2% to 11% for questions in which missing data were observed. To account for missing data in our primary analysis we employed multiple imputation using 10 imputations. All reported parameter estimates accounted for the multiple imputation [24]. To assess the sensitivity of the resulting estimates to the imputation procedure, we also performed a complete-case analysis and found the estimates to be consistent with those obtained using multiple imputation.

We conducted secondary analyses to explore potential associations between the main reason for participants enrolling (personal benefit, benefit of mankind, benefit of future generations, or doctor’s suggestion) and willingness to participate. We fitted a proportional odds model to each scenario, including the reason to participate variable. Given the exploratory nature of the analyses, we only considered complete cases.

All analyses were conducted using R version 3.2.2. Imputations were performed using the Hmisc package. Kappa statistics and Cronbach’s alpha statistics were calculated using the psych package in R.

## Results

Ninety-one participants completed the survey: 22 with a clinical diagnosis of dementia, 32 with MCI, and 37 with normal cognition. One respondent’s consensus diagnosis was cognitive impairment not meeting criteria for dementia or MCI. In analyses assessing attitudes towards trial participation, this patient was included as part of the MCI group. The participant was excluded from analyses of self-reported and consensus diagnosis agreement. Table 1 describes the demographic characteristics for the study respondents stratified by clinical diagnosis. The MCI group had an equal number of males and females, while the cognitively normal and dementia groups had more males. Most participants were white (91.1%). The mean age of the respondents was 73.5 years for those with dementia, 69.9 years for MCI, and 72.5 years for the cognitively normal. Dementia participants tended to be more educated. The majority of participants were married and most lived with a spouse, though the proportion living with a spouse was highest among the dementia group. As expected, most cognitively normal and MCI participants functioned independently, while the majority of dementia participants required assistance with basic or instrumental activities of daily living. Similarly, most cognitively normal and MCI participants reported completing the survey on their own (80.6% and 80.7%, respectively), while a majority of dementia participants reported receiving assistance from their study partner on more than half the questions or completing the survey together with their study partner (90.9%).

**Table 1:** Characteristics of survey respondents

Participant characteristics	Consensus diagnosis		
	CN (*n* = 37)	MCI (*n* = 32)	Dementia (*n* = 22)
Age, mean (SD)	72.46 (10.4)	69.94 (9.5)	73.55 (11.4)
Education, mean (SD)	17.22 (1.9)	16.78 (2.7)	19.73 (17.9)
Female sex, *n* (%)	16 (43.2%)	16 (50.0%)	6 (27.3%)
Race, *n* (%)			
White	35 (97.2%)	29 (90.6%)	18 (81.8%)
African-American	0 (0.0%)	3 (9.4%)	1 (4.6%)
Asian	1 (2.8%)	0 (0.0%)	2 (9.1%)
Other	0 (0.0%)	0 (0.0%)	1 (4.6%)
Hispanic, *n* (%)	0 (0.0%)	1 (3.1%)	1 (4.6%)
Marriage status, *n* (%)			
Married	25 (67.6%)	22 (68.8%)	17 (77.3%)
Widowed	6 (16.2%)	1 (3.1%)	3 (13.6%)
Divorced	3 (8.1%)	6 (18.8%)	2 (9.1%)
Never married	2 (5.4%)	3 (9.4%)	0 (0.0%)
Living as married	1 (2.7%)	0 (0.0%)	0 (0.0%)
Partner involvement, *n* (%)			
No help	29 (80.6%)	25 (80.7%)	2 (9.1%)
Less than half	5 (13.9%)	1 (3.2%)	0 (0%)
More than half	2 (5.6%)	5 (16.1%)	13 (59.1%)
Together	0 (0.0%)	0 (0.0%)	7 (31.8%)
Living situation, *n* (%)			
Lives alone	7 (18.9%)	8 (25.0%)	3 (13.6%)
Lives with spouse or partner	22 (59.5%)	20 (62.5%)	16 (72.7%)
Lives with relative or friend	2 (5.4%)	0 (0.0%)	1 (4.6%)
Lives with group	4 (10.8%)	1 (3.1%)	1 (4.6%)
Other	2 (5.4%)	3 (9.4%)	1 (4.6%)
Independence, *n* (%)			
Able to live independently	36 (97.3%)	31 (96.9%)	8 (36.4%)
Requires some assistance with complex activities	0 (0.0%)	1 (3.1%)	12 (54.6%)
Requires some assistance with basic activities	1 (2.7%)	0 (0%)	1 (4.5%)
Completely dependent	0 (0.0%)	0 (0%)	1 (4.5%)

“Partner Involvement” refers to how much the study partner assisted the patient in completing the survey

“Independence” refers to how well participants can perform daily activities

*CN* cognitively normal, *MCI* mild cognitive impairment, *SD* standard deviation

Participants were asked to identify their diagnosis in the context of how they would complete the survey. Discrepancies between self-reported and ADRC consensus diagnoses were observed in each group (Table 2). Concordance rates were 73% for the dementia group, 48% for the MCI group, and 84% for the cognitively normal group. The weighted kappa statistic was 0.71 (95% confidence interval (CI) 0.58–0.84) indicating substantial agreement, while the unweighted kappa statistic was 0.52 (95% CI 0.36–0.67) indicating moderate agreement [25]. Table 2 provides the number of participants in each category.

**Table 2:** Self-reported versus ADRC consensus diagnosis^a^

Consensus diagnosis (*n*)	Self-reported diagnosis		
	CN (*n*)	MCI (*n*)	Dementia (*n*)
CN	26	5	0
MCI^b^	13	13	1
Dementia	2	4	16

^a^Limited to participants with both clinical and self-reported diagnoses available

^b^Subject with cognitive impairment not due to MCI was excluded from this analysis

Unweighted kappa statistic 0.52, 95% confidence interval (CI) 0.36–0.67

Weighted kappa statistic 0.71, 95% CI 0.58–0.84

*ADRC* Alzheimer’s Disease Research Center, *CN* cognitively normal, *MCI* mild cognitive impairment

Table 3 displays the group responses to questions related to the type of treatment under study and the procedures often involved in AD clinical trials. Participants within each diagnostic group were most willing to participate in trials of approved medications and trials that involved MRI. Table 4 shows the responses of the groups to questions about visit frequency and trial length; while willingness was highest for studies with annual visits, no trends relating to trial length were apparent.

**Table 3:** Responses to survey questions regarding willingness to participate in clinical trials including various treatments and procedures

Trial design elements	Diagnostic group	Likelihood of participation		
		Very unlikely (score = 1–2)	Neutral (score = 3–5)	Very likely (score = 6–7)
Approved	CN	6 (16.2%)	19 (51.4%)	12 (32.4%)
	MCI	2 (6.25%)	12 (37.5%)	18 (56.3%)
	Dementia	0 (0.00%)	5 (22.7%)	17 (77.3%)
Experimental	CN	11 (29.7%)	21 (56.8%)	5 (13.5%)
	MCI	4 (12.5%)	17 (53.1%)	11 (34.4%)
	Dementia	5 (22.7%)	4 (18.2%)	13 (59.1%)
Pill	CN	7 (18.9%)	21 (56.8%)	9 (24.3%)
	MCI	4 (12.5%)	14 (43.8%)	14 (43.8%)
	Dementia	4 (18.2%)	3 (13.6%)	15 (68.2%)
Infused	CN	12 (32.4%)	23 (62.2%)	2 (5.4%)
	MCI	9 (28.1%)	17 (53.1%)	6 (18.8%)
	Dementia	6 (27.3%)	9 (40.9%)	7 (31.8%)
Diet/exercise	CN	4 (10.8%)	18 (48.65%)	15 (40.5%)
	MCI	4 (12.5%)	10 (31.3%)	18 (56.3%)
	Dementia	3 (13.6%)	8 (36.4%)	11 (50.0%)
Supplement	CN	4 (10.8%)	13 (35.1%)	20 (54.1%)
	MCI	2 (6.3%)	9 (28.1%)	21 (65.6%)
	Dementia	2 (9.1%)	8 (36.4%)	12 (54.6%)
MRI	CN	7 (18.9%)	12 (32.4%)	18 (48.7%)
	MCI	7 (21.9%)	8 (25.0%)	17 (53.1%)
	Dementia	4 (18.2%)	9 (40.9%)	9 (40.9%)
PET scan	CN	12 (32.4%)	11 (29.7%)	14 (37.8%)
	MCI	8 (25.0%)	9 (28.1%)	15 (46.8%)
	Dementia	4 (18.2%)	9 (40.9%)	9 (40.9%)
Lumbar	CN	24 (64.8%)	12 (32.4%)	1 (2.7%)
punctures	MCI	15 (46.9%)	9 (28.1%)	8 (25.0%)
	Dementia	10 (45.4%)	9 (40.9%)	3 (13.6%)
Bleeding in brain or gut	CN	30 (81.1%)	6 (16.2%)	1 (2.7%)
	MCI	18 (56.3%)	11 (34.4%)	3 (9.4%)
	Dementia	10 (45.5%)	9 (40.9%)	3 (13.6%)
Headache or nausea	CN	15 (40.5%)	20 (54.1%)	2 (5.4%)
	MCI	9 (28.1%)	16 (50.0%)	7 (21.9%)
	Dementia	9 (40.9%)	8 (36.4%)	5 (22.7%)

Values are shown as *n* (%)

*CN* cognitively normal, *MCI* mild cognitive impairment, *MRI* magnetic resonance imaging, *PET* positron emission tomography

**Table 4:** Responses to survey questions regarding willingness to participate in clinical trials of various lengths and frequency of visits

Trial design elements	Diagnostic group	Likelihood of participation		
		Very unlikely (score = 1–2)	Neutral (score = 3–5)	Very likely (score = 6–7)
Annual visits	CN	5 (13.5%)	14 (37.8%)	18 (48.7%)
	MCI	2 (6.3%)	5 (15.6%)	25 (78.1%)
	Dementia	5 (22.7%)	1 (4.6%)	16 (72.7%)
Monthly visits	CN	13 (35.1%)	16 (43.2%)	8 (21.6%)
	MCI	3 (9.4%)	14 (43.8%)	15 (46.9%)
	Dementia	6 (27.3%)	4 (18.2%)	12 (54.6%)
Weekly visits	CN	17 (45.9%)	17 (45.9%)	3 (8.1%)
	MCI	10 (31.3%)	15 (46.9%)	7 (21.9%)
	Dementia	10 (45.5%)	6 (27.3%)	6 (27.3%)
1-year study	CN	8 (21.6%)	15 (40.5%)	14 (37.8%)
	MCI	3 (9.4%)	11 (34.4%)	18 (56.3%)
	Dementia	5 (22.7%)	4 (18.2%)	13 (59.1%)
2-year study	CN	7 (20.0%)	19 (54.3%)	9 (25.7%)
	MCI	4 (13.8%)	8 (27.6%)	17 (58.6%)
	Dementia	5 (25.0%)	3 (15.0%)	12 (60.0%)
5-year study	CN	11 (30.6%)	17 (47.2%)	8 (22.2%)
	MCI	6 (18.8%)	11 (34.4%)	15 (46.9%)
	Dementia	7 (31.8%)	3 (13.6%)	12 (54.6%)

Values are shown as *n* (%)

*CN* cognitively normal, *MCI* mild cognitive impairment

**Fig. 1:**
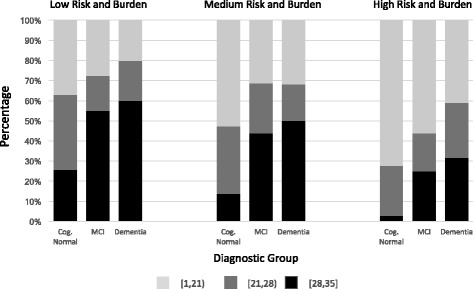
Group scores for trial composites. Diagnostic group summary scores for the composite scenarios are illustrated. *Light gray* = composite score of 1–21 (low willingness), *dark gray* = composite score 21–28 (moderate willingness), *black* = composite score 28–35 (high willingness). *Cog.* cognitively, *MCI* mild cognitive impairment

To compare the willingness of the diagnostic groups to participate, we created three composite scenarios of various risk and burden levels: low, medium, and high. Two trends were evident from the composite scenarios (Fig. 1). First, within each composite scenario, more severe diagnosis was associated with greater willingness to participate (dementia > MCI > cognitively normal). Second, with greater risk and burden, willingness to enroll was reduced for each diagnostic group. In the low-risk, low-burden scenario there was a high proportion of willing participants for each diagnostic group (63% of cognitively normal, 72% of MCI, and 80% of dementia). In the high-risk, high-burden scenario 28% of cognitively normal participants, 44% of MCI, and 59% of dementia participants were highly willing to participate.

**Fig. 2:**
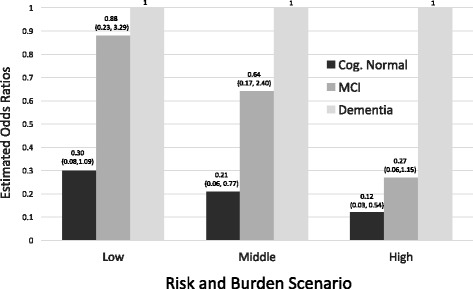
Relative willingness estimates. Proportional odds regression estimates (95% CI) for “high willingness” compared to the dementia diagnostic group are presented for the cognitively normal (*Cog. Normal*) and mild cognitive impairment (*MCI*) groups, stratified by level of trial risk and burden (low, middle, and high scenarios)

To more formally characterize willingness to participate among the three diagnostic groups, we fitted a proportional odds model adjusting for potential confounding factors (Table 5 and Fig. 2). The adjusted model confirmed the overall observations related to the diagnostic groups. The estimated odds of “high” versus “low” willingness comparing MCI patients to dementia patients ranged from 0.27 to 0.88, while the estimated odds of “high” versus “low” willingness comparing cognitively normal patients to dementia patients ranged from 0.12 to 0.30. Odds ratios for gender, marital status, age, and education were similar for each composite scenario (Table 5). Women were twice as likely to be willing compared to men; married participants were three times as likely as unmarried; for every 5 years of age the odds of being highly willing increased 14–30%; and higher education was associated with a roughly 20% reduction in the odds of being highly willing to participate.

**Table 5:** Estimated odds ratios for each scenario based on the proportional odds model

Covariate	Low risk, low burden		Medium risk, medium burden		High risk, high burden	
	Estimated OR (95% CI)	*P* value	Estimated OR (95% CI)	*P* value	Estimated OR (95% CI)	*P* value
Diagnosis						
Dementia	1.0		1.0		1.0	
MCI	0.88 (0.23–3.29)	0.844	0.64 (0.17–2.40)	0.507	0.27 (0.06–1.15)	0.076
CN	0.30 (0.08–1.09)	0.067	0.21 (0.06–0.77)	0.019	0.12 (0.03–0.54)	0.006
Partner involvement						
Some help	0.65 (0.22–1.96)	0.448	1.12 (0.38–3.30)	0.834	0.58 (0.16–2.11)	0.408
Marital status						
Married (living as)	3.06 (1.12–8.35)	0.029	2.26 (0.82–6.22)	0.114	1.84 (0.63–5.41)	0.266
Gender						
Female	2.04 (0.84–4.97)	0.117	1.99 (0.80–4.95)	0.139	2.64 (0.97–7.17)	0.056
Age (5 years)	1.30 (1.04–1.62)	0.021	1.21 (0.96–1.51)	0.102	1.14 (0.90–1.45)	0.276
Education	0.78 (0.65–0.93)	0.007	0.77 (0.64–0.93)	0.007	0.80 (0.65–0.97)	0.024

*CI* confidence interval, *CN* cognitively normal, *MCI* mild cognitive impairment, OR odds ratio

**Fig. 3:**
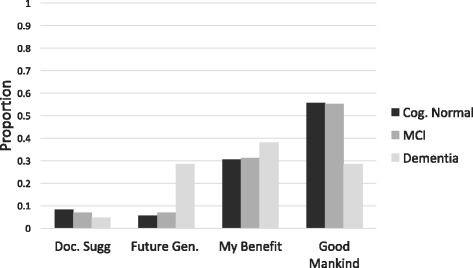
Participant reasons for enrolling. The proportion of respondents that chose each response as their main reason for enrolling in a clinical trial are presented, stratified by diagnostic group. *Cog.* Cognitively, *Doc.* doctor, *Gen.* generation, *MCI* mild cognitive impairment

We also asked participants to state their main reason for enrolling. Figure 3 shows the proportions of patients within each diagnostic group that gave each response for a trial of an experimental medication. Thirty-eight percent of dementia patients said their main reason for enrolling was for their own benefit, 29% said it was for the good of mankind, and 29% said it was for future generations. A majority of MCI and cognitively normal participants responded that their main reason for enrolling was for the good of mankind (57% and 54%, respectively). The patterns of responses were identical when participants were asked their reason for enrolling in a trial of an approved therapy (data not shown).

In secondary analyses, we investigated whether the primary motivation for enrolling was associated with composite willingness scores in the three scenarios. We performed a likelihood ratio test to determine whether this covariate should be included in each of the models. We failed to reject the null hypothesis that the main reason for enrolling is associated with the composite score in the low- and high-risk, high-burden scenarios. The test statistics and *p* values were 6.10 (*p* = 0.11) in the low-risk, low-burden scenario, and 6.13 (*p* = 0.11) in the high-risk, high-burden scenario. In the medium-risk, medium-burden scenario, there was sufficient evidence to reject the null hypothesis (test statistic = 9.54, *p* value = 0.02), indicating an association between the primary motivation for enrolling and willingness to participate in trials.

## Discussion

The results of this study indicate that the simple adoption of AD dementia trial design features in predementia trials may result in challenging trial recruitment. These recruitment challenges may be most evident in trials that involve more invasive assessment methods (such as PET or lumbar puncture) or more invasive treatments (such as infusions or vaccines). For example, the odds of high willingness to enroll in a low-risk, low-burden trial for MCI participants were estimated to be 12% less (0.88, 95% CI 0.23–3.29) than that of dementia participants. For a high-risk, high-burden trial, however, the odds of high willingness to participate were estimated to be 73% less (0.27, 95% CI 0.06–1.15). The odds of high willingness to participate for cognitively normal participants were estimated to be 70% (0.30, 95% CI 0.08–1.09), 79% (0.21, 95% CI 0.06–0.77) and 88% (0.12, 95% CI 0.03–0.54) less than those of the dementia group in low-, medium-, and high-risk and -burden composite scenarios, respectively.

The dementia group also differed from the MCI and cognitively normal groups in the reported rationale for participating in trials. Whereas altruism was the most common reason for considering enrollment in MCI and cognitively normal participants, personal benefit was the most common reason for those with dementia (Fig. 3). These results are in line with previous studies aiming to elucidate AD trial decision-making. AD is a debilitating and deadly disease; gaining access to a new therapy that may improve the health of the AD dementia patient is an important motivating factor in trial enrollment decisions, which are made in partnership between the AD patient and their caregiver [10, 14, 16, 26, 27]. In contrast, cognitively normal participants and MCI patients may emphasize altruism as a primary rationale for enrollment and may make trial decisions more unilaterally [20, 21]. In fact, in this study 80% of MCI participants reported completing the survey without input from their study partner and less than 50% of these participants correctly self-reported their diagnosis, with most incorrectly reporting as being cognitively normal (Table 2). This may suggest that these participants did not perceive a need for personal benefit of enrolling in trials. While understanding patient rationale for enrollment may provide important guidance for designing recruitment strategies, we found no relationship between the rationale for participating and willingness to enroll for two out of three composite scenarios, regardless of diagnostic category.

Some factors did predict willingness to enroll. Females were more likely than males, older age was associated with greater willingness, and married participants were 84–306% more likely to be willing to enroll compared to unmarried participants, depending on the diagnostic group. The latter finding is noteworthy given the striking pattern of overrepresentation of spousal dyads in AD dementia trials [28], suggesting that attitudes [29] rather than eligibility [30] may drive these enrollment patterns.

There are limitations to this study. Data were collected from participants in a longitudinal research study performed at an academic medical center. Most participants were Caucasian, married, and had high levels of education. This may limit generalizability to the general population, but also may recapitulate the sample biases known to occur in AD trials [28, 31]. The survey had a relatively low response rate, potentially limiting the generalizability even to the research-friendly population from which the sample is derived. Because the survey was sent to a cross-sectional selection of a time-varying cohort, we are unable to obtain demographic data on the individuals who did not respond to the survey. This limits our ability to compare the characteristics of responders and nonresponders. We excluded people who had previously participated in clinical trials; future studies should examine how previous trial participation affects attitudes toward enrollment. Our small sample size limited the power to detect differences between the three diagnosis groups. Participants are likely to have had different preferences and weightings in regards to trial characteristics and in their interpretation of the Likert scale. Future studies would benefit from incorporating these into the analysis. The paper survey was completed in the home, and the reliability of participant reporting of the involvement of the study partner is unknown. This may be particularly relevant to the MCI participants, who self-reported their own diagnosis incorrectly 50% of the time. It is possible that greater involvement of the study partner could have reduced this error rate, or could have changed the responses to other survey items. How MCI patients approach the decision whether to participate in AD trials is largely unknown. We chose to analyze the survey data based on the consensus diagnosis of the participants, rather than their self-reported diagnosis. This decision was based on the means by which these groups would be recruited to trials and the trials for which they would be eligible. We acknowledge, however, that failure of patients to recognize their own eligibility for a trial based on the diagnostic category in which it is being conducted (e.g., MCI patients who self-report as cognitively normal may ignore MCI trial invitations as not appropriate for them) could be a critical barrier to recruitment.

The study was based on hypothetical questions about trials rather than actual enrollment decisions, and failed to address some important aspects of modern AD trials, notably including the need for biomarker testing and disclosure in preclinical and prodromal trials. The biomarker disclosure process is complex and challenging to address in a paper survey [32]. This, and the desire to send identical instruments to all diagnostic groups, led us to focus on trial aspects that are more consistent across diagnostic groups.

## Conclusions

Recruitment for Alzheimer’s disease clinical trials is often a difficult task. If trials are burdensome, participants and their study partners are less likely to enroll. We found that in exemplary trial scenarios, the willingness to participate was greater for dementia patients compared to MCI and cognitively normal participants. These results suggest that AD dementia clinical trial designs may encounter even more difficult recruitment when used for prodromal and preclinical AD studies. Moreover, these results indicate the need for planning studies to ensure recruitment feasibility for each diagnostic category. Major international efforts to create cohorts that are “trial ready” may address some of these challenges [33–35], but a more thorough understanding of which design elements encourage participation in each potential study population will allow researchers to carefully design trials in ways that optimize recruitment and ensure trial success.

## Additional file

Additional file 1: Appendix. (DOC 59 kb)

## Abbreviations

AD: Alzheimer’s disease
ADRC: Alzheimer’s Disease Research Center
CI: Confidence interval
MCI: Mild cognitive impairment
MRI: Magnetic resonance imaging
OR: Odds ratio
PET: Positron emission tomography
UCLA: University of California Los Angeles
