# Supplementary material for: Attitudes toward clinical trials across the Alzheimer’s disease spectrum
Source: Alzheimers Res Ther. 2017 Oct 4;9:81. doi: 10.1186/s13195-017-0311-5 (PMC5628443; doi:10.1186/s13195-017-0311-5)
Supplement: Additional file 1: — Appendix. (DOC 59 kb) [file 13195_2017_311_MOESM1_ESM.doc]

**Questionnaire**

First, we need to get some basic information from you.

Your Name (please print):­­­­­­­­­ _________________________________________________________________

Date completing the survey (month / day / year): ______________________________________

Date of birth (month / day / year): ______________________________________

The decision to participate in a clinical trial is often supported by a loved one (spouse, partner, child, or friend). In fact, most clinical trials related to Alzheimer’s disease require the participant to bring another person (called a “study partner”) with them to study visits. Similarly, the UCLA Easton Center longitudinal study (in which you are a participant) requires each person to have a study partner. You may wish to complete this survey with your study partner.

The name of your study partner in the Easton Center longitudinal study (please print):

_______________________________________________________________________________________

Please check the box that most accurately represents the level of involvement by your study partner when completing this survey. [Please check only one box and feel free to come back and answer this question at the end]

🞎_1_ I completed the survey without anyone else’s input

🞎_2_ I asked my study partner’s input on less than half of the questions

🞎_3_ I asked my study partner’s input on more than half of the questions

🞎_4_ My study partner and I completed the survey together as a team

🞎_5_ I completed the survey on behalf of my loved one who is a participant in the UCLA study

The purpose of this survey is to better understand your feelings about clinical trials. As you fill out this questionnaire, we ask you to think about the idea of participating in clinical trials *in general,* rather than thinking about specific trials you may have heard about on television or at the Easton Center. Consider instead, the things that would or would not make you likely to participate in a research study of a new treatment, such as a drug.

If you are a participant in the UCLA Easton Center Longitudinal study because you have been diagnosed with dementia, we ask that you and your study partner consider a clinical trial that aims to examine a medication’s ability to slow the progression of dementia. If you are a participant in the UCLA study because you have been diagnosed with Mild Cognitive Impairment (MCI), we ask that you consider a trial that aims to examine a medication’s ability to improve memory and lower the risk for getting dementia. If you are a participant in the UCLA study as a cognitively normal control subject, we ask that you consider a clinical trial that aims to examine a medication’s ability to prevent Alzheimer’s disease and dementia.

Based on this description, please tell us which of the following best describes the way you (and your study partner) will approach the survey?

🞎_1_ Considering a clinical trial of a medication to slow the progression of dementia (I have been diagnosed with dementia)

🞎_2_ Considering a clinical trial of a medication to lower the risk of getting dementia (I have been diagnosed with mild cognitive impairment)

🞎_3_ Considering a clinical trial of a medication to prevent Alzheimer’s disease (I participate in the Longitudinal study as a cognitively normal control)

Please check the box that best represents the level at which you agree

1=Disagree very much, 7=Agree very much -or- 1=Extremely unlikely, 7=Extremely likely

I would consider participating in a clinical trial of an approved medication.

Disagree Somewhat Somewhat Agree

Very much Disagree Disagree Neutral Agree Agree Very much

🞎_1_ 🞎_2_ 🞎_3_ 🞎_4_ 🞎_5_ 🞎_6_ 🞎_7_

I would be likely to enroll in a clinical trial of an approved medication.

Disagree Somewhat Somewhat Agree

Very much Disagree Disagree Neutral Agree Agree Very much

🞎_1_ 🞎_2_ 🞎_3_ 🞎_4_ 🞎_5_ 🞎_6_ 🞎_7_

If I enrolled in a study of an approved medication, part of my reason for doing so would be to help science and medicine/the good of mankind.

Disagree Somewhat Somewhat Agree

Very much Disagree Disagree Neutral Agree Agree Very much

🞎_1_ 🞎_2_ 🞎_3_ 🞎_4_ 🞎_5_ 🞎_6_ 🞎_7_

If I enrolled in a study of an approved medication, part of my reason for doing so would be hope that the medicine would be of benefit to me.

Disagree Somewhat Somewhat Agree

Very much Disagree Disagree Neutral Agree Agree Very much

🞎_1_ 🞎_2_ 🞎_3_ 🞎_4_ 🞎_5_ 🞎_6_ 🞎_7_

If I enrolled in a study of an approved medication, part of my reason for doing so would be to help my children/future generations.

Disagree Somewhat Somewhat Agree

Very much Disagree Disagree Neutral Agree Agree Very much

🞎_1_ 🞎_2_ 🞎_3_ 🞎_4_ 🞎_5_ 🞎_6_ 🞎_7_

If I enrolled in a study of an approved medication, part of my reason for doing so would be if my doctor told me it was a good idea.

Disagree Somewhat Somewhat Agree

Very much Disagree Disagree Neutral Agree Agree Very much

🞎_1_ 🞎_2_ 🞎_3_ 🞎_4_ 🞎_5_ 🞎_6_ 🞎_7_

If I enrolled in a study of an approved medication, the most important reason for doing so would be (PLEASE CHECK ONLY ONE ANSWER)

🞎 To help science and medicine; the good of mankind.

🞎 The hope that the medicine would be of benefit to me.

🞎 To help my children/future generations.

🞎 If my doctor told me it was a good idea

I would consider participating in a clinical trial of an experimental medication.

Disagree Somewhat Somewhat Agree

Very much Disagree Disagree Neutral Agree Agree Very much

🞎_1_ 🞎_2_ 🞎_3_ 🞎_4_ 🞎_5_ 🞎_6_ 🞎_7_

I would be likely to enroll in a clinical trial of an experimental medication.

Disagree Somewhat Somewhat Agree

Very much Disagree Disagree Neutral Agree Agree Very much

🞎_1_ 🞎_2_ 🞎_3_ 🞎_4_ 🞎_5_ 🞎_6_ 🞎_7_

If I enrolled in a study of an experimental medication, part of my reason for doing so would be to help science and medicine/ the good of mankind.

Disagree Somewhat Somewhat Agree

Very much Disagree Disagree Neutral Agree Agree Very much

🞎_1_ 🞎_2_ 🞎_3_ 🞎_4_ 🞎_5_ 🞎_6_ 🞎_7_

If I enrolled in a study of an experimental medication, part of my reason for doing so would be hope that the medicine would be of benefit to me.

Disagree Somewhat Somewhat Agree

Very much Disagree Disagree Neutral Agree Agree Very much

🞎_1_ 🞎_2_ 🞎_3_ 🞎_4_ 🞎_5_ 🞎_6_ 🞎_7_

If I enrolled in a study of an experimental medication, part of my reason for doing so would be to help my children/future generations.

Disagree Somewhat Somewhat Agree

Very much Disagree Disagree Neutral Agree Agree Very much

🞎_1_ 🞎_2_ 🞎_3_ 🞎_4_ 🞎_5_ 🞎_6_ 🞎_7_

If I enrolled in a study of an experimental medication, part of my reason for doing so would be if my doctor told me it was a good idea.

Disagree Somewhat Somewhat Agree

Very much Disagree Disagree Neutral Agree Agree Very much

🞎_1_ 🞎_2_ 🞎_3_ 🞎_4_ 🞎_5_ 🞎_6_ 🞎_7_

If I enrolled in a study of an experimental medication, the most important reason for doing so would be

🞎 To help science and medicine/the good of mankind.

🞎 The hope that the medicine would be of benefit to me.

🞎 To help my children/future generations.

🞎 If my doctor told me it was a good idea

If a clinical trial of an experimental medication included possible risks for side effects such as bleeding in the brain or gut, how likely would you be to participate?

Extremely Somewhat Somewhat Extremely

Unlikely Unlikely Unlikely Neutral Likely Likely Likely

🞎_1_ 🞎_2_ 🞎_3_ 🞎_4_ 🞎_5_ 🞎_6_ 🞎_7_

If a clinical trial of an experimental medication included possible risks for side effects such as headache or nausea, how likely would you be to participate?

Extremely Somewhat Somewhat Extremely

Unlikely Unlikely Unlikely Neutral Likely Likely Likely

🞎_1_ 🞎_2_ 🞎_3_ 🞎_4_ 🞎_5_ 🞎_6_ 🞎_7_

If the clinical trial of an experimental medication required weekly study visits at UCLA, how likely would you be to participate?

Extremely Somewhat Somewhat Extremely

Unlikely Unlikely Unlikely Neutral Likely Likely Likely

🞎_1_ 🞎_2_ 🞎_3_ 🞎_4_ 🞎_5_ 🞎_6_ 🞎_7_

If the clinical trial of an experimental medication required monthly study visits at UCLA, how likely would you be to participate?

Extremely Somewhat Somewhat Extremely

Unlikely Unlikely Unlikely Neutral Likely Likely Likely

🞎_1_ 🞎_2_ 🞎_3_ 🞎_4_ 🞎_5_ 🞎_6_ 🞎_7_

If the clinical trial of an experimental medication required annual study visits at UCLA, how likely would you be to participate?

Extremely Somewhat Somewhat Extremely

Unlikely Unlikely Unlikely Neutral Likely Likely Likely

🞎_1_ 🞎_2_ 🞎_3_ 🞎_4_ 🞎_5_ 🞎_6_ 🞎_7_

If a clinical trial of an experimental medication was 12 months long (length of the entire study), how likely would you be to participate?

Extremely Somewhat Somewhat Extremely

Unlikely Unlikely Unlikely Neutral Likely Likely Likely

🞎_1_ 🞎_2_ 🞎_3_ 🞎_4_ 🞎_5_ 🞎_6_ 🞎_7_

If a clinical trial of an experimental medication was 2 years long (length of the entire study), how likely would you be to participate?

Extremely Somewhat Somewhat Extremely

Unlikely Unlikely Unlikely Neutral Likely Likely Likely

🞎_1_ 🞎_2_ 🞎_3_ 🞎_4_ 🞎_5_ 🞎_6_ 🞎_7_

If a clinical trial of an experimental medication was 5 years long (length of the entire study), how likely would you be to participate?

Extremely Somewhat Somewhat Extremely

Unlikely Unlikely Unlikely Neutral Likely Likely Likely

🞎_1_ 🞎_2_ 🞎_3_ 🞎_4_ 🞎_5_ 🞎_6_ 🞎_7_

How likely would you be to participate in a clinical trial of a life style change like diet or exercise?

Extremely Somewhat Somewhat Extremely

Unlikely Unlikely Unlikely Neutral Likely Likely Likely

🞎_1_ 🞎_2_ 🞎_3_ 🞎_4_ 🞎_5_ 🞎_6_ 🞎_7_

How likely would you be to participate in a clinical trial of a vitamin or dietary supplement?

Extremely Somewhat Somewhat Extremely

Unlikely Unlikely Unlikely Neutral Likely Likely Likely

🞎_1_ 🞎_2_ 🞎_3_ 🞎_4_ 🞎_5_ 🞎_6_ 🞎_7_

How likely would you be to participate in a clinical trial of an experimental medication in the form of a pill?

Extremely Somewhat Somewhat Extremely

Unlikely Unlikely Unlikely Neutral Likely Likely Likely

🞎_1_ 🞎_2_ 🞎_3_ 🞎_4_ 🞎_5_ 🞎_6_ 🞎_7_

How likely would you be to participate in a clinical trial of an experimental medication that was infused into the blood stream?

Extremely Somewhat Somewhat Extremely

Unlikely Unlikely Unlikely Neutral Likely Likely Likely

🞎_1_ 🞎_2_ 🞎_3_ 🞎_4_ 🞎_5_ 🞎_6_ 🞎_7_

How likely would you be to participate in a clinical trial of an experimental medication that was injected into the brain?

Extremely Somewhat Somewhat Extremely

Unlikely Unlikely Unlikely Neutral Likely Likely Likely

🞎_1_ 🞎_2_ 🞎_3_ 🞎_4_ 🞎_5_ 🞎_6_ 🞎_7_

How likely would you be to participate in a clinical trial of an experimental medication that required magnetic resonance imaging (MRI) scans (MRIs take pictures of the brain using a large magnet, are loud, and can make some people feel claustrophobic)?

Extremely Somewhat Somewhat Extremely

Unlikely Unlikely Unlikely Neutral Likely Likely Likely

🞎_1_ 🞎_2_ 🞎_3_ 🞎_4_ 🞎_5_ 🞎_6_ 🞎_7_

How likely would you be to participate in a clinical trial of an experimental medication that required positron emission tomography (PET) scans (PET scans take pictures of the brain using radiation exposure)?

Extremely Somewhat Somewhat Extremely

Unlikely Unlikely Unlikely Neutral Likely Likely Likely

🞎_1_ 🞎_2_ 🞎_3_ 🞎_4_ 🞎_5_ 🞎_6_ 🞎_7_

How likely would you be to participate in a clinical trial of an experimental medication that required lumbar punctures (LP; also known as “spinal taps” are procedures to remove cerebrospinal fluid from the spinal canal)?

Extremely Somewhat Somewhat Extremely

Unlikely Unlikely Unlikely Neutral Likely Likely Likely

🞎_1_ 🞎_2_ 🞎_3_ 🞎_4_ 🞎_5_ 🞎_6_ 🞎_7_

How likely would you be to participate in a clinical trial of an experimental medication that required bringing a study partner to every visit?

Extremely Somewhat Somewhat Extremely

Unlikely Unlikely Unlikely Neutral Likely Likely Likely

🞎_1_ 🞎_2_ 🞎_3_ 🞎_4_ 🞎_5_ 🞎_6_ 🞎_7_

Financial incentive would increase the likelihood that I would choose to participate in a clinical trial of an experimental medication.

Disagree Somewhat Somewhat Agree

Very much Disagree Disagree Neutral Agree Agree Very much

🞎_1_ 🞎_2_ 🞎_3_ 🞎_4_ 🞎_5_ 🞎_6_ 🞎_7_

If some of the visits could be conducted in my home, it would make me more likely to participate in a clinical trial of an experimental medication.

Disagree Somewhat Somewhat Agree

Very much Disagree Disagree Neutral Agree Agree Very much

🞎_1_ 🞎_2_ 🞎_3_ 🞎_4_ 🞎_5_ 🞎_6_ 🞎_7_

If I knew that we were going to get the actual medication and not placebo, it would make me more likely to participate in a clinical trial of an experimental medication.

Disagree Somewhat Somewhat Agree

Very much Disagree Disagree Neutral Agree Agree Very much

🞎_1_ 🞎_2_ 🞎_3_ 🞎_4_ 🞎_5_ 🞎_6_ 🞎_7_

If I knew that we were going to get the placebo and not actual medication, it would make me more likely to participate in a clinical trial of an experimental medication.

Disagree Somewhat Somewhat Agree

Very much Disagree Disagree Neutral Agree Agree Very much

🞎_1_ 🞎_2_ 🞎_3_ 🞎_4_ 🞎_5_ 🞎_6_ 🞎_7_

**Final Checklist**

____ Include your name and your study partner’s name on page 1?

____ Did you answer each question only once, including the question on page 1?

____ Include all nine (9) pages in the returned materials?
